# Variation in wood physical properties and effects of climate for different geographic sources of Chinese fir in subtropical area of China

**DOI:** 10.1038/s41598-021-83500-w

**Published:** 2021-02-25

**Authors:** Ren You, Ninghua Zhu, Xiangwen Deng, Jing Wang, Fei Liu

**Affiliations:** 1 Faculty of Life Science and Technology, Central South University of Forestry and Technology, Changsha, 410004 Hunan Province China; 2 Faculty of Forestry, Central South University of forestry and Technology, Changsha, 410004 China; 3 National Engineering Laboratory for Applied Technology of Forestry & Ecology in South China, Changsha, 410004 China; 4 Huitong National Field Station for Scientific Observation and Research of Chinese Fir Plantation Ecosystem in Hunan Province, Huitong, 438107 China

**Keywords:** Forest ecology, Plant ecology

## Abstract

Chinese fir is one of the most important commercial timber species in China, with many geographic sources. However, little is known of the variation in wood physical properties among them. To explore the differences in wood physical properties and their influencing factors, five geographic sources of Chinese fir were selected. The variance inflation factor, stepwise regression, and principle component analysis were used to reduce multicollinearity and dimensions of the 19 wood physical properties (including density, shrinkage, and mechanical properties). The results showed that the wood density differed significantly among five geographic sources. The tangential shrinkage rate and radial shrinkage rate reached maximum values in black-heart Chinese fir (HNYX-T) but accompanied by the lowest value for difference dry shrinkage. The wood density and mechanical properties of HNYX-T was exceeded to that of others geographic sources. Fast-growth Chinese fir (FJYK-P) had the lowest value for all mechanical properties. The precipitation and temperature had significant correlations with the wood physical properties of this five geographic sources. The temperature in summer was mainly positive correlated with physical properties, while precipitation was negatively correlated with them. HNYX-T had the highest comprehensive score of PCA, followed by JXCS-R, emerged as higher-quality geographic source, which is important for selecting and utilizing geographic sources in forest management.

## Introduction

Wood is the basic component of root and stem tissues of woody plants^1–3^. The physical properties of wood govern many important links in the growth and development of woody plants, such as water transport and mechanical support, which are closely related to the individual morphological structure of trees, life history strategies, interspecific resource competition, community dynamics, and even terrestrial ecosystem functioning^4–6^. The wood physical properties characteristics mainly include its density, dry shrinkage coefficient elasticity, and strength, to name a few^2^, and they vary with tree species and life forms^7^. For example, conifers usually have lower density, while hardwood trees have higher density^8^. Wood’s physical properties are closely related to the intrinsic factors of the trees, like the growth process of trees, for example, fast growing trees with lower wood density, and they are significantly affected by extrinsic factors, such as climate factors^9^. Furthermore, studying the physical properties of wood and identifying its influencing factors can help to better understand the structure and function of terrestrial ecosystems and thereby also improve our ability to assess and predict the response of forests to global climate change^10^.

There are many researchers compared the density between different timbers, which showed that different wood species have different density^11,12^. Wood density is a characteristic of high genetic heritability, which is a resource can be strongly influenced by genetics and the wood production potential that a site presents is largely represented by the affinity of its genetic material^13,14^. There is a large body of research on wood density. For example, Sadaaki compared the variation in density of young and mature wood of *Chamaecypariso btusa, Cryptomeria japonica* and *Pinus densiflora*^15^. Chen studied the wood density and fiber morphology of new clones of poplar tree named ‘*Qinbaiyang*’ and also analyzed the differences between four varieties of it and compared their respective physical properties advantages^16^. Eitaro et al. discussed the relationship between the timing of late wood formation and the genetic variation of wood density in *Larix kaempferi*^17^. In addition, researchers have also reported on interspecific and intraspecific variation in drought susceptibility of *Abies*, whose relationships to wood density and growth traits were summarized^18^. Finally, the timber department of the Wood Industry Research Institute of the Chinese Academy of Forestry conducted a study which compared the wood physical properties of main tree species in China including Chinese fir (*Cunninghamia lanceolata* (Lamb) Hook)^19^.

Those previous studies mainly focused on the density of wood and overlooked the possible influence for merchantable timber quality of other important wood properties. This precludes a comprehensive evaluation of wood properties, since many wood properties are known co-vary and interact with each other to regulate a tree's hydraulic conductivity, mechanical support, storage of nutrients and water, and growth and senescence^20^. In particular, kinds of shrinkage are basic physical properties, whose association with wood moisture fluctuations constitutes an important piece of information for the wood-processing industry^21^, as it can affect the use of individual species of wood, and even the properties of wood value-added wood products^22^. The bending strength is the most important mechanical property studied and the compression strength parallel to the grain of wood and tensile strength parallel to the grain are also the important physical properties indexes, which usually serve as the basis for selecting better timber components^23^. Therefore, in this context, it is arguably not enough to simply evaluate wood physical properties by wood density alone^24–26^.

Chinese fir (*Cunninghamia lanceolata* (Lamb) Hook) is one of the most valuable and best-known subtropical timber species, currently occupying about 25% of the plantations in subtropical areas of China^27^ and provides up to 30% of the harvested logs for China's timber industry^28^. And it is also the main economic tree species in northern Vietnam^29^. This tree has high use-value in private houses due to its suite of characteristics, namely fast growth, high yield, beautiful wood texture, resistance to insects (like termites^30^) and belong to corrosion-resistant material, as well as its adaptability to arid and barren habitats^31^. Chinese fir is widely used in furniture, construction, shipbuilding and paper making and other fields, so it has a high economic value^32^. Large-scale planting Chinese fir can increase the forest coverage rate to prevent soil and water flow loss phenomenon, protecting the ecological balance at the same time, to a certain extent, the local soil quality and air quality improved, and create a good environment that occupy the home to local residents^33^. As the dominant fast-growing cultivated timber species in southern China, this conifer in different geographical regions shows some differences due to the long-term influence of the local geographical environment and lineage divergence^34^. The different geographic sources of Chinese fir can be distinguished by unique characteristics, such as variant of Chinese fir wood with red-heart wood can produce more attractive and high market value wood^35^, with better physicomechanical properties^36^. Therefore, it is of great significance to study geographical variation Chinese fir’s physical properties to develop its potential as material for decorative and furniture wood panels and to increase its added value^37^. Concerning the breeding of Chinese fir trees, inevitably the trend to choose those varieties with excellent growth and wood physical properties quality^38^. Here, 19 physical properties of Chinese fir were comprehensively analyzed, to (1) compare the variation in wood physical properties among five geographic sources, (2) explore their influencing factors, (3) evaluate the five geographic sources by using a comprehensive PCA (principal component analysis) score—all of which can figure prominently in selecting and utilizing the high-quality varieties of this tree.

## Results

### Variation in wood density

**Figure 1:**
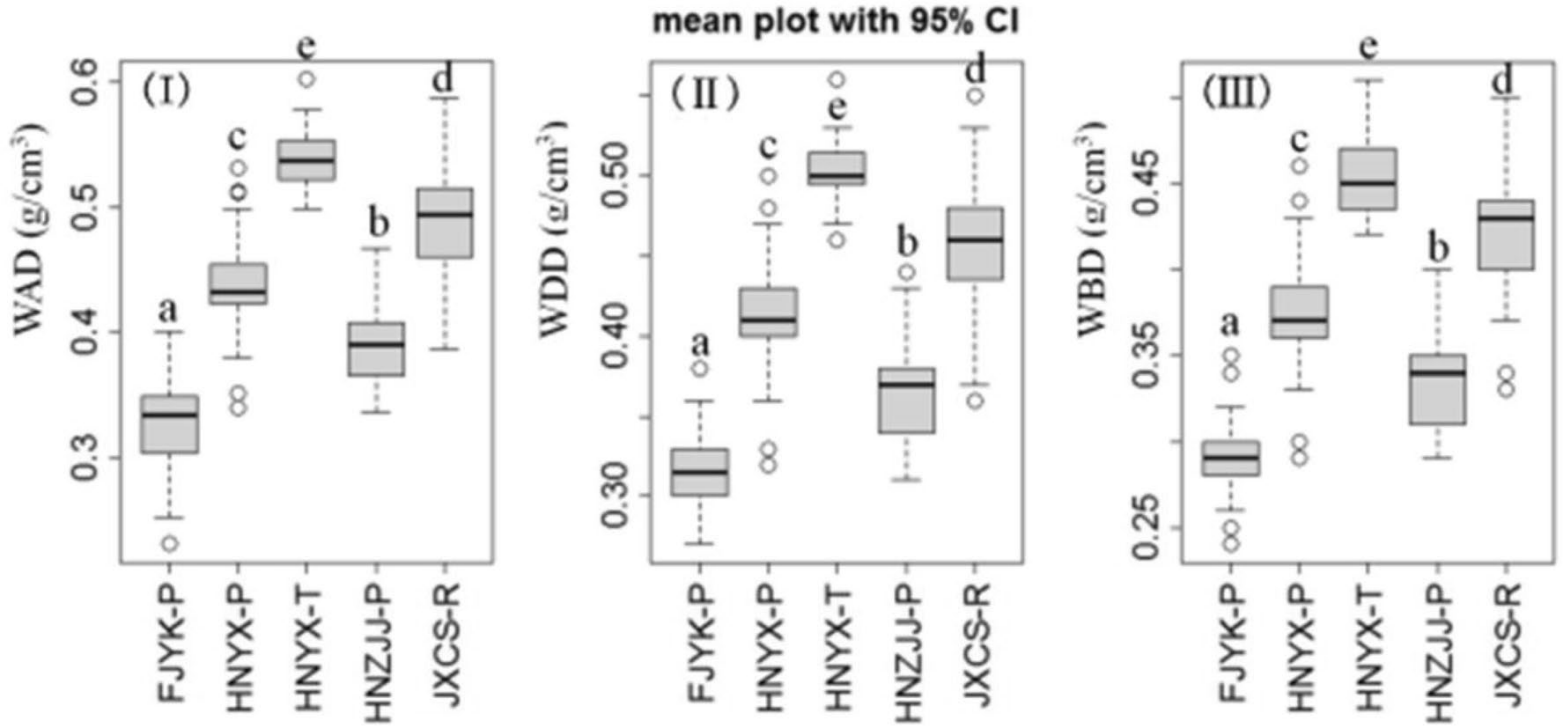
There kinds of wood density in different Chinese fir geographic sources, (**I**) is the wood air-dry density (WAD g/cm^3^); (**II**) is the wood all-dried density (WDD g/cm^3^); (**III**) is the wood basic density (WAD g/cm^3^). Different letters (a, b, c, d, e) mean significant difference at 0.05 level.

The values of Chinese fir’s wood physical properties varied considerably among different geographic sources and Tukey-HSD testing showed that some of these differences were statistically significant (Fig. 1). The maximum value (HNYX-T) of wood all-dry density (WDD) was 62.70% higher than the minimum (FJYK-P). The WDD of each source was consistent with the classification and performance indexes of conifer trees in the timber strength grade for structural use, a standard in China’s forestry industry^39^: FJYK-P was at level S10 (< 0.30 g/cm^3^) and HNYX-T was at level S36 (< 0.50 g/cm^3^).

The wood air-dried density (WAD) results matched the WDD, in that significant differences were found among all the geographic sources, with maximum value (HNYX-T) 60.85% higher than the minimum (FJYK-P) (Fig. 1a,b). The maximum value (HNYX-T) was 60.85% higher than the minimum value (FJYK-P). According to the classification regulations of wood quality in China^12^, the WAD of fast-growing Chinese fir was at the lowest level (≤ 0.35 g/cm^3^). Likewise, according to the classification standard of physical properties indicators: FJYK-P was at level I (0.35 g/cm^3^), while the other four geographic sources were at level II (0.35–0.55 g/cm^3^). Through many experimental studies^19^, the Chinese Academy of Forestry concluded the WAD of Chinese fir in various regions ranged from 0.32 to 0.42 g/cm^3^. But here we found the HNYX-T (0.54 g/cm^3^) and JXCS-R (0.49 g/cm^3^) values exceeded 0.45 g/cm^3^.

The wood basic density (WBD) of HNYX-T (0.46 g/cm^3^) and JXCS-R (0.42 g/cm^3^) were highest among the five geographic sources, being lowest for FJYK-P (0.29 g/cm^3^), though HNYX-P (0.37 g/cm^3^) was similar HNZJJ-P (0.34 g/cm^3^) (Fig. 1c). The maximum value of HNYX-T was 63% higher than the FJYK-P (0.25 g/cm^3^). In terms of classification standards for physical mechanical indexes^40^, FJYK-P belonged to level I (≤ 0.30 g/cm^3^), HNYX-T belonged to level III (0.46–0.60 g/cm^3^), and the rest of geographic sources belonged to level II (0.31–0.45 g/cm^3^).

### Variation in shrinkage

Among the five geographic sources from four sampled sites, the most represented in shrinkage was that of black-heart Chinese fir. According to Table 1, the tangential shrinkage rate of air-dry **(**TSR.RD) of HNYX-T’s wood was 3.41% and it was lowest in FJYK-P (1.06%). Radial shrinkage rate of air-dry **(**RSR.RD) of JXCS-R (1.20%) and HNYX-T (1.60%) was higher in JXCS-R (1.20%) and HNYX-T (1.60%) than in FJYK-P (0.08%).

**Table 1:** The statistical analysis of shrinkage (air-dry) of Chinese fir.

Sources	TSR.RD	RSR.RD	DDS.RD	VSR.RD
FJYK-P	1.06 ± 0.57	0.08 ± 0.36	8.74 ± 5.06	1.27 ± 0.95
HNYX-P	2.66 ± 0.80	0.98 ± 0.38	2.78 ± 0.74	3.76 ± 1.05
HNYX-T	3.41 ± 0.84	1.60 ± 0.52	2.30 ± 0.66	5.12 ± 1.28
HNZJJ-P	1.66 ± 0.61	0.55 ± 0.48	2.64 ± 0.92	2.29 ± 1.04
JXCS-R	2.68 ± 1.21	1.20 ± 0.37	2.47 ± 22.33	3.89 ± 1.09

Data are means ± SE.

*TSR.RD* Tangential shrinkage rate of air-dry density, *RSR.RD* Radial shrinkage rate of air-dry density, *DDS.RD* Difference dry shrinkage of air-dry density, *VSR.RD* Volume shrinkage rate of air-dry density.

The volume shrinkage rate of air-dry *(*VSR.RD) was ranked as follows: FJYK-P < HNZJJ-P < HNYX-P < JXCS-R < HNYX-T. The rank ordering of DDS.RD (air-dry) differed slightly: HNZJJ-P < HNYX-T < JXCS-R < HNYX-P < FJYK-P. Among the five sources, the maximum difference dry shrinkage of air-dry (DDS.RD) value (HNYX-T) was 62.10% higher than the lowest (FJYK-P). According to China’s timber classification regulations: the drying shrinkage rate of FJYK-P was at the lowest level (≤ 2.5%), while the rest of the Chinese fir sources were at the moderate level (2.6–4.0%). VSR.RD of HNYX-T was intermediate level (4.6–5.5%), the rest were low-grade (≤ 4.5%).

Wood dry shrinkage was also an important indicator for evaluating its physical properties. The tangential shrinkage rate of all-dry (TSR.LD) of HNYX-P was the highest among the five geographic sources. The radial shrinkage rate of all-dry (RSR.LD) of HNYX-T was 67.70% higher than that of FJYK-P (2.10%). Among the five sources, the highest volume shrinkage rate of all-dry (VSR.LD) was obtained for HNYX-P, whereas the DDS.LD was the greatest in FJYK-P (2.85%), and was the lowest in JXCS-R (1.97%) (Table 2).

**Table 2:** The statistical analysis of shrinkage (all-dry ) of Chinese fir.

Sources	TSR.LD	RSR.LD	DDS.LD	VSR.LD
FJYK-P	5.80 ± 0.91	2.1 ± 0.58	2.85 ± 0.44	8.18 ± 1.32
HNYX-P	6.60 ± 1.29	2.79 ± 0.70	2.42 ± 0.38	9.61 ± 1.73
HNYX-T	5.82 ± 1.39	3.10 ± 0.96	1.98 ± 0.45	9.35 ± 2.02
HNZJJ-P	5.76 ± 1.03	2.61 ± 0.76	2.27 ± 0.41	8.64 ± 1.62
JXCS-R	4.95 ± 1.00	2.63 ± 0.47	1.97 ± 0.59	7.91 ± 1.07

Data are means ± SE.

*TSR.LD* Tangential shrinkage rate of all-dry density, *RSR.LD* Radial shrinkage rate of all-dry density, *DDS.LD* Difference dry shrinkage of all-dry density, *VSR.LD* Volume shrinkage rate of all-dry density.

### Variation in mechanical properties

As Table 3 shows, the modulus of rupture **(**MOR) of the five geographic sources was ranked as follows: JXCS-R > HNYX-T > HNYX-P > HNZJJ-P > FJYK-P. The flexural strength index was determined according to the China’s classification standard of physical properties indexes. Both HNYX-T (110.70 MPa) and JXCS-R (95.60 MPa) were categorized as level III (88.10–118.00 Mpa); all other fir sources were designated level II (54.10–88.10 Mpa).

**Table 3:** The statistical analysis of wood mechanical properties of Chinese fir.

Sources	MOE (MPa)	MOR (MPa)	TSG (MPa)	CSG (MPa)
FJYK-P	8639.42 ± 1375.67	63.09 ± 11.48	55.64 ± 14.94	32.60 ± 6.63
HNYX-P	11,870.34 ± 1791.81	89.85 ± 13.96	94.06 ± 22.59	48.92 ± 4.31
HNYX-T	10,587.36 ± 1699.75	93.34 ± 25.86	127.64 ± 38.98	56.33 ± 4.93
HNZJJ-P	9945.92 ± 1373.69	71.45 ± 18.58	78.90 ± 22.36	41.01 ± 7.49
JXCS-R	10,901.91 ± 1151.05	96.66 ± 7.41	105.39 ± 15.68	50.52 ± 4.02

Data are means ± SE.

*MOE* modulus of elasticity (MPa), *MOR* modulus of rupture (MPa), *TSG* tensile strength parallel to grain (MPa), *CSG* compression strength parallel to the grain (MPa).

The modulus of elasticity *(*MOE) of HNYX-P was highest among the five geographic sources. Their ranking for tensile strength parallel to grain (TSG) was HNYX-T > JXCS-R > HNYX-P > HNZJJ-P > FJYK-P, for which the maximum was 47.60% higher than the minimum value. According to the grading standard of mechanical properties, HNYX-T, JXCS-R, and HNYX-P qualified for level III (10.4–13.2 GPa), while the other two sources were at level II (7.5–10.3 GPa).

The compression strength parallel to the grain (CSG) had this ranking: HNYX-T > JXCS-R, HNYX-P > HNZJJ-P > FJYK-P, for which the maximum 58.0% higher than the minimum value. According to the wood grading standards in the grain compression index, HNZJJ-P and FJYK-P were at level II (29.1–44.0 MPa) and the rest of geographic sources were at level III (44.1–59.0 MPa) (Table 3).

The compression strength perpendicular to the grain of total tensile (CPG.TT) among geographic sources was ranked as follows: HNYX-T > JXCS-R > HNYX-P > HNZJJ-P > FJYK-P (Table 4). Its maximum value (HNYX-T) was 29.3% higher than the minimum (FJYK-P). The ranking for compression strength perpendicular to the grain of total radial (CPG.TR) was slightly different: HNYX-T > JXCS-R > HNYX-P > HNZJJ-P > FJYK-P, for which the maximum was 42.1% higher than the minimum value. Compression strength perpendicular to the grain of part radial (CPG.PR) had the same rank order as CPG.TT, with a maximum value (HNYX-T) 35.0% higher than the minimum (FJYK-P). Finally, compression strength perpendicular to the grain of part tensile (CPG.PT) was ranked as HNYX-T > JXCS-R > HNZJJ-P > HNYX-P > FJYK-P for the five geographic sources of Chinese fir.

**Table 4:** The statistical analysis of wood mechanical properties of Chinese fir.

Sources	CPG.TT	CPG.TR	CPG.PT	CPG.PR
FJYK-P	3.92 ± 1.29	3.29 ± 0.78	14.22 ± 2.56	14.00 ± 4.30
HNYX-P	7.33 ± 1.01	5.13 ± 1.42	24.03 ± 6.62	17.05 ± 4.59
HNYX-T	13.29 ± 1.35	8.15 ± 1.91	41.64 ± 6.23	28.11 ± 5.96
HNZJJ-P	5.63 ± 1.94	4.76 ± 2.11	18.52 ± 8.87	17.59 ± 10.02
JXCS-R	9.34 ± 0.80	7.22 ± 0.41	29.20 ± 2.91	25.52 ± 1.83

Data are means ± SE.

*CPG.TT* compression strength perpendicular to the grain (total tensile) (MPa), *CPG.TR* compression strength perpendicular to the grain (total radial) (MPa), *CPG.PT* compression strength perpendicular to the grain (part tensile) (MPa), *CPG.PR* compression strength perpendicular to the grain (part radial) (MPa).

### Factors influencing wood physical properties

#### Climate factors effect on wood physical properties

**Figure 2:**
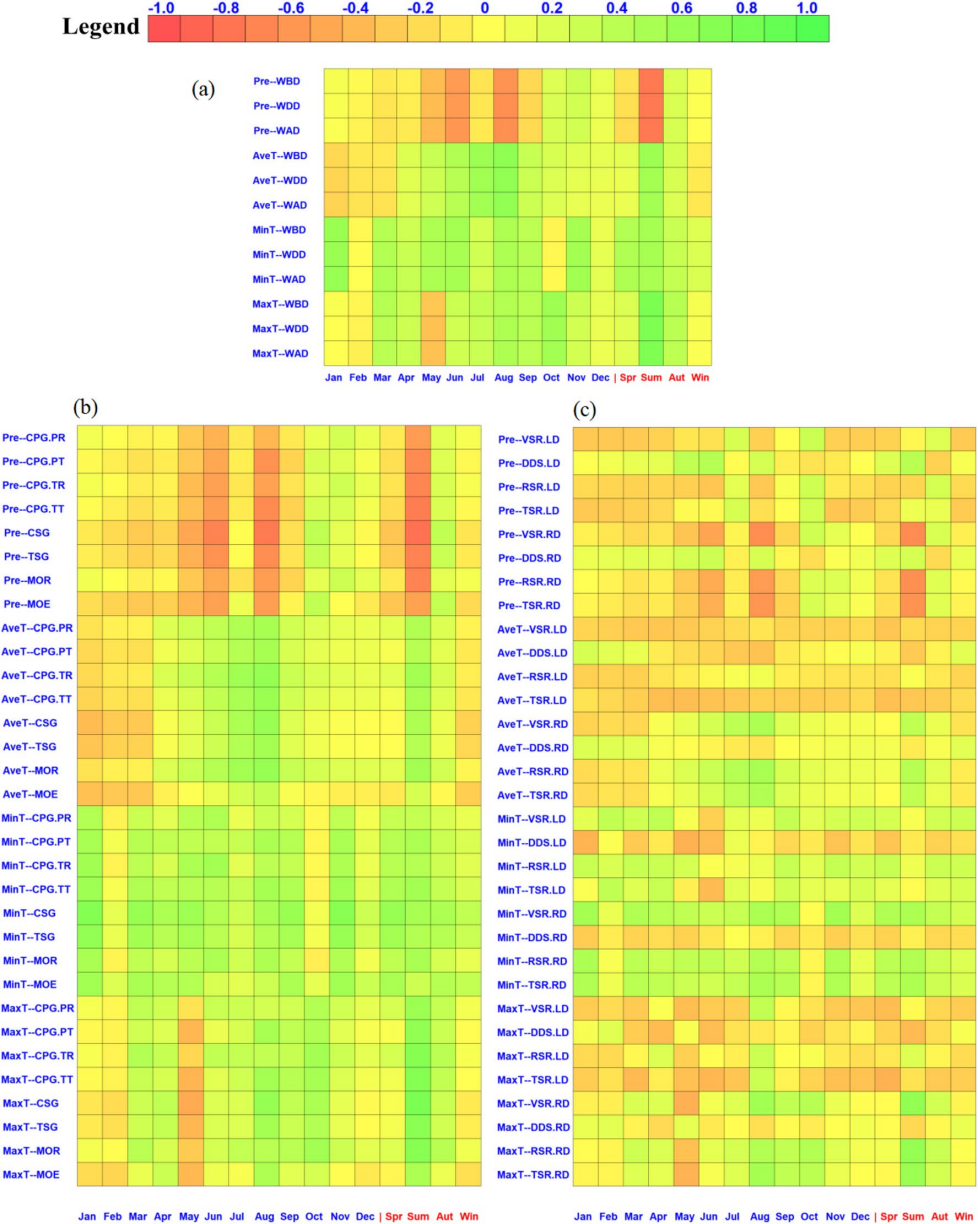
The correlation between climate and wood physical properties, and (**a**) is the wood density; (**b**) is the mechanical properties and (**c**) is the shrinkage. Pre is sum precipitation of every month. AveT is average daily mean temperature of each month. MinT is average daily min temperature of each month. MaxT is average daily max temperature of each month. Spr is the value of Mar, Apr, May. Sum is the value of Jun, Jul, Aug. Aut is the value of Sep, Oct, Nov. Win is the value of Dec, Jan, Feb.

The influence of precipitation on the three kinds of density was consistent. Pre in January, October, November, and December was positively related to wood density, while it was negatively correlated with density in others months, especially in May (*r* = − 0.39), June (*r* = − 0.59), and August (*r* = − 0.64). On a seasonal scale, Pre in summer was negatively correlated with density (*r* = − 0.77), but it was positively correlated with autumn (*r* = 0.22). MaxT was positively correlated with density during the whole year, except in May (*r* = − 0.34), and likewise with wood density but most strongly in summer (*r* = 0.75). MinT was positively correlated with density, especially in Jan (*r* > 0.7), though it was not significantly so in February and October (*r* < − 0.01). AveT was positively correlated with density except in January, February, and March, reaching statistical significance in June (*r* = 0.42), July (*r* = 0.55), and August (*r* = 0.64). AveT was positively correlated with density in all seasons except winter (*r* = − 0.12) (Fig. 2a).

Pre was positively correlated with TSR.RD, RSR.RD, DDS.RD, and VSR.RD in October, November, and December. Pre was significant negatively correlated with TSR.RD, RSR.RD, VSR.RD in June, August, and summer (*r* > 0.45). Pre showed no significant correlation with TSR.LD, RSR.LD, DDS.LD, and DDS.RD, whose correlation coefficients were 0.1–0.3. But Pre was negatively correlated with VSR.LD most of the year (except July, October). AveT was negatively correlation with TSR.RD, RSR.RD, and VSR.RD in January, February, March, and winter; however, AveT showed no significant correlation with DDS.RD. AveT was negatively correlated with TSR.LD, RSR.LD, DDS.LD, and VSR.LD during the whole year. In general, MinT had a significant positive relationship to TSR.RD (*r* = 0.47), RSR.RD (*r* = 0.48), and VSR.RD (*r* = 0.52), except in October, and it was negatively correlated with DDS.RD. MinT was positively related to RSR.LD, VSR.LD, yet negative related to DDS.LD. MaxT was negatively correlated with TSR.RD, RSR.RD, VSR.RD in January, February, May, and December, and winter. MaxT showed no significant correlation with DDS.RD, RSR.LD, DDS.LD or VSR.LD (Fig. 2c).

Pre had significant negative correlations with all of the mechanical properties in May, June, August, and summer, as evince by Fig. 2b, which also showed positive correlations in October. As we can seen, the effects of Pre on wood density and mechanical properties have the same tendency. Pre in all other months was not significantly correlated with mechanical properties (*r* < 0.3). AveT in January, February, March, and winter was negatively correlated with mechanical properties, but was positively correlated with mechanical properties in June, July, and summer, when the correlation coefficient reached its maximum, in August (*r* = 0.67). MinT was significantly correlated with mechanical properties, which was strongest in January (*r* > 0.75), while it was showed no significant correlation in Feb and Oct (*r* < 0.2). On a seasonal scale, MinT in winter was showed no significant correlation with mechanical properties (*r* < 0.3). As for MaxT, which was positively correlation with mechanical properties in August, October, and summer, while it was negatively correlation with mechanical properties in May, which was a interesting result we got from Fig. 2b. MinT showed a significant correlation with CSG, whose coefficient was higher 0.75 in summer.

#### PCA analysis of physical properties

Although the physical properties of wood can be affected by all 19 variables (including WBD WDD WAD MOE MOR TSG CSG CPG.TT CPG.TR CPG.PT CPG.PR TSR.RD RSR.RD DDS.RD VSR.RD TSR.LD RSR.LD DDS.LD and VSR.LD) considered, it was not necessary to include all these variables in our research. Variance inflation factor was used to judge whether collinearity exists among the variables. We calculated the VIF values of all 19 variables. Among them, WAD (83.63), WDD (6196.39), WBD (6015.66), TSR.RD (13.93), RSR.RD (11.36), VSR.RD (22.57), VSR.RD (22.57), TSR.LD (38.35), RSR.LD (44.30), DDS.LD (16.49), VSR.LD (123.78) had VIF values > 10. Those of WDD and WBD were > 1000. Through stepwise regression modeling, 14 variables without multicollinearity were retained (i.e., MOE, MOR, TSG, CSG, CPG.TT, CPG.TR, CPG.PT, CPG.PR, DDS.RD, WDD, DDS.LD, TSR.RD, RSR.RD, VSR.LD).

**Figure 3:**
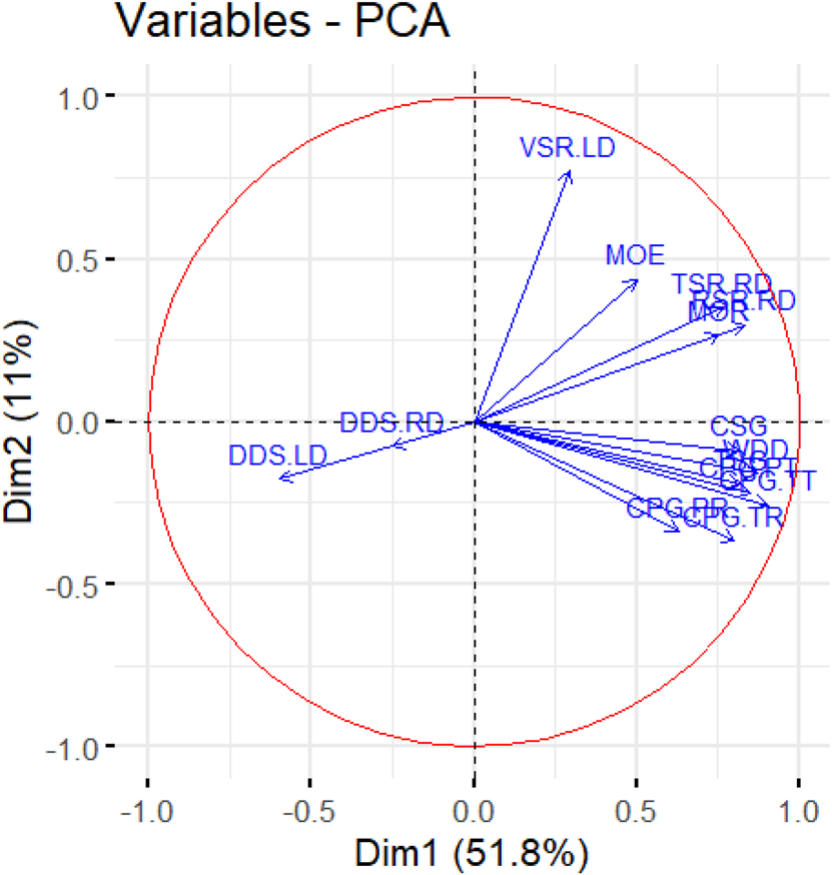
Sequence diagram plot of PCA analysis showing the relationship among physical properties of wood.

**Figure 4:**
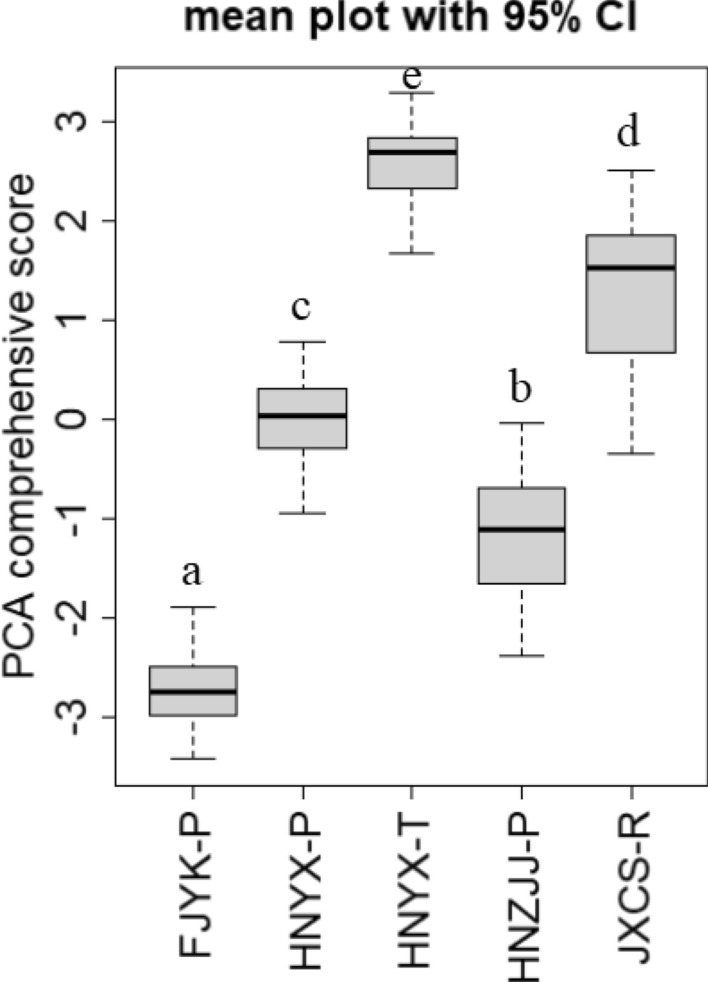
Mean comprehensive score of PCA plot with 95% CI. Different letters (a, b, c, d, e) mean significant difference at 0.05 level.

PCA was applied to the above 14 selected physical variables. These results showed that the physical properties of wood loaded strongly on the first axis of the PCA, explaining 51.8% of variation in the 14 tested properties, while the second axis explained 11.0% of it. MOE, MOR, TSR.RD, RSR.RD, and VSR.LD loaded on the positive axis of PC1 and PC2. Both DDS.LD and DDS.RD loaded on the negative axis of PC1 and PC2, while TSG, CSG, CPG.TT, CPG.TR, CPG.PT, CPG.PR, and WDD loaded on the positive axis of PC1 and the negative axis of PC2 (Fig. 3). For a comprehensive evaluation of Chinese fir’s wood physical properties, we calculated the comprehensive scores of five geographic sources via the PCA. In this respect, significant differences were detected among the five geographic sources. Among them, the comprehensive score of HNYX-T was the highest whereas that of FJYK-P was the lowest (Fig. 4).

## Discussion

### Variation in physical properties

There were significant differences in wood density among the five geographic sources (*P* < 0.05). This was an expected result, and consistent with studies carried out elsewhere. For example, Luo et al. found that the wood density and mechanical properties of 32 Chinese fir clones differed significantly among them^36^, and wood density varies across globally among tropical tree species^41^. Importantly, the WBD values between 0.29 and 0.54 g/cm^3^ in our study fell within the range (0.11–1.39 g/cm^3^) reported for 2456 tropical forest tree species^42^. However, the average WBD (0.37 g/cm^3^) in our study was lower than that (0.57 g/cm^3^) found for trees from two neotropical rain forests and seven subtropical tree species in China^43^. Compared with other species, Chinese fir is well known for its fast growth, which may explain its rather low density^7^.

FJYK-P was the fastest growth geographic source in this study, perhaps because it has the lowest wood density, and shrinkage volumetric and radial values, and tangential and volumetric shrinkage coefficients. Wood geographic sources whose trees are of high-density harbor more dimensional variation, because they produce more wood per unit volume^44,45^. The mean value of volumetric shrinkage for the wood of Bracatinga (*Mimosa scabrella Benthan*) was 7.65%^46^, which is more than the values we obtained for Chinese fir woods. The eucalyptus tree (*Eucalyptus benthamii Matenet Cambage*) had radial shrinkage, tangential shrinkage, and an anisotropy coefficient of 5.91%, 13.87%, and 2.36^47^, respectively, which were almost the same as those found for the fir of five geographic sources in our study.

Through mechanical experiments carried out on 16 Chinese fir samples, Cai et al. found that in addition to the transverse compressive strength, the flexural strength, flexural elastic modulus, impact toughness, shear strength, and splitting strength were all higher than the radial surface, which also confirmed the accuracy of our conclusion^48^. The mean values of MOE, MOR, and CSG of fir from the five geographic sources in our study (Tables 3 and 4) were similar to those found in a previous study for *L. sibirica* that grows naturally in Mongolia, yet higher than those for *L. kaempferi* planted in Japan^49,50^. But the mechanical properties of HNYX-T are greater than other softwood species^51^.

The physical properties of wood determine its post-harvest applications. Among the five geographic sources, the comprehensive score was highest for black-heart Chinese fir, whose wood is generally used in the construction of ships, bridges, and as construction material^52^. Red-heart Chinese fir had many advantages, such as its round, straight trunk and fine grain, and being tough and corrosion resistant, with a large proportion of red heart wood, all of which generally make it popular in buildings and the furniture market^8^. Due to its growth rate and relatively low physical properties, fast-growing Chinese fir is generally marketable and widely in demand. We can see that the practical implications of Chinese fir’s utilization were consistent with our wood property measurements.

### Control factors for physical properties

Trees and the climate factors are interdependent and are continually interacting. The growth and development of trees cannot be distinguished from the influence from climate conditions^53,54^. Our study showed that precipitation was significantly and negatively correlated with wood density in spring and summer (Fig. 2a). The possible reason for this finding is that abundant rainfall in summer causes trees to grow faster, resulting in a decreased density of formed wood, which is in line with a previous study^12^. In considering temperature only, a suitably high temperature can promote the growth of trees, but when that temperature limit is exceeded, it will inhibit the growth of trees.

The present study also revealed a significant and positive correlation of temperature with Chinese fir’s mechanical properties (Fig. 2b). Similarly, according to Zhang et al.'s research, after eliminating the influence of evolutionary effects, the physical properties of wood was marked by a significant positive correlation with temperature. A positive correlation between temperature and MOE was also reported for *Populus deltoides Marsh*^50^.

## Conclusion

By sampling trees and considering environmental factors, here we compared the wood physical properties of five different fir-growing geographic sources and the climate effects on physical properties. Our results showed that a certain degree of independence exists among the 19 wood physical properties. After calculated the comprehensive scores of five geographic sources via PCA, the HNYX-T emerged as the geographic source of highest-quality timber quality, which is important when evaluating geographic sources to select and utilize for growing Chinese fir. The influence of temperature was mainly positive with respect to physical properties of Chinese fir trees while precipitation was negatively correlated with them. But there were many additional factors likely affecting Chinese fir’s physical properties, which could be promising for further research. Through the PCA of physical properties, the comprehensive score differed significantly among the five geographic sources, with HNYX-T having the highest score, followed by JXCS-R, HNYX-P, HNZJJ-P, and FJYK-P. The results provide a theoretical basis for future timber production and applications in forest management in subtropical China.

## Materials and methods

### Site description

Five geographic sources of Chinese fir samples in China were investigated: the fast growth Chinese fir samples were selected in Yangkou, Fujian Province (FJYK-P); the normal Chinese fir samples were selected in Zhangjiajie, Hunan Province (HNZJJ-P); the red-heart Chinese fir of samples were selected in Chenshan, Jiangxi province (JXCS-R); the black-heart Chinese fir (HNYX-T) and: normal Chinese fir (HNYX-P) samples were selected in the same county, which located in Yongshun, Hunan Province. All five sites are located in a subtropical region that has a moderate climate throughout the year and receives ample rainfall. During the year, high temperatures and much rainfall in these five areas are concentrated from June to September, while low temperatures and little rain begin in November and end in the following January (Table 5).

**Table 5:** The geographical and climate conditions of sampling sites.

Sources	Sn	Area	County	Pro	SA (year)	SD (N.hm^-2^)	Long. (E)	Lat. (N)	Alt (m)	MAT (°C)	MAP (mm)
FJYK-P	30	Yangkou forest farm	Nanping	Fujian	53	385	117°53′	26°49′	570	18.5	1880
HNYX-T	20	Xiaoxi town	Yongshun	Hunan	53	360	110°15′	28°48′	662	11.5	1350
HNYX-P	48	Xiaoxi town	Yongshun	Hunan	52	584	110°15′	28°45′	600	11.5	1350
HNZJJ-P	20	Wulingyuan district	Zhangjiajie	Hunan	52	776	110°26′	29°18′	866	16.6	1400
	25	Cili district	Zhangjiajie	Hunan	52	615	110°53′	29°14′	550	16.7	1390
JXCS-R	55	Chenshan forest farm	Anfu	Jiangxi	51	429	114°24′	27°30′	710	17.7	1553

*Sn* is the number of test material samples, *Pro* is the name of province, *SA* is the average age of stand, *SD* is the stand density, *Long* is longitude, *Lat* is latitude, *Alt* is altitude, *MAT* is mean daily average temperature, *MAP* is the mean annual precipitation.

### Sample collection

**Figure 5:**
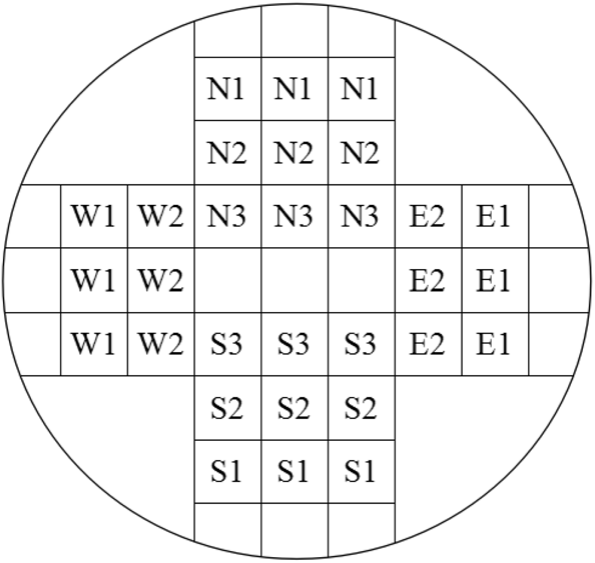
The sketch map of sawing sample.

At spring, we selected 3–5 trees from each of five source areas in 2019. The average age of these trees was 50–52 years. To measure the 19 wood properties, we selected samples spanning from the breast height 1.3–3.3 m along bole. Following the Chinese government document *GB/T 1929-2009*^55^ for the sawing of test material samples for analyzing wood physical properties, 40 mm × 40 mm test strips were uniformly intercepted along the horizontal plane in the parts lying outside the pulp center (Fig. 5). There are 198 test strips as our samples (including 30 samples of FJYK-P, 20 samples of HNYX-T, 48 samples of HNYX-P, 45 samples of HNZJJ-P, and 55 samples of JXCS-R) according to Eq. (1).

1 $$ n = \frac{{V^{2} t^{2} }}{{P^{2} }} $$

where n is the number of samples (N), V is the coefficient of variation, t is the reliability index, P is the accuracy index.

**Figure 6:**
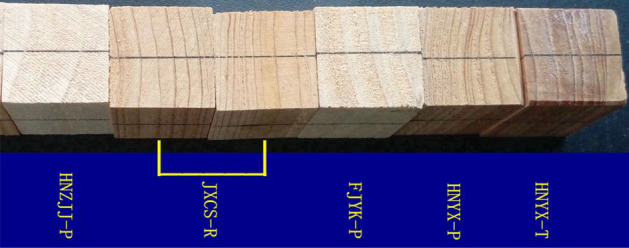
The cross-section photo of samples.

The modulus of rupture and modulus of elasticity of the collected specimens, as well as their compression strength parallel to the grain, density, compression strength, and dry shrinkage test specimen, compression strength perpendicular to the grain (total tensile and total radial) and to the grain specimens (part tensile and part radial), and tensile strength parallel to the grain specimens were intercepted of blank in each one (Fig. 6).

### Experimental methods and data source

The values of wood basic density (WBD, g/cm^3^), wood all-dry density (WDD, g/cm^3^), wood air-dried density (WAD, g/cm^3^), modulus of elasticity (MOE, MPa), modulus of rupture (MOR, MPa), tensile strength parallel to the grain (TSG, MPa), compression strength parallel to the grain (CSG, MPa), compression strength perpendicular to the grain (CPG, MPa), tangential shrinkage rate (TSR, MPa), radial shrinkage rate (RSR), difference dry shrinkage (DDS), and volume shrinkage rate (VSR), size of the specimens were strips with 40 mm × 40 mm, which selected in north–south direction and east–west direction specimens with dry-treated at 50 °C. All determined as described in government documents of China: *GB/T 1932-2009*, *GB/T 1933-2009*, *GB/T 1933-2009*, *GB/T 1939-2009*, *GB/T 1936.1-2009*, G*B/T 1936.2-2009*^56–61^. Measurements of the mechanical properties were carried out on the MWD-50 (micro-controlled wood universal tester, China). All the experiments were conducted in the wood research laboratory of the Central South University of Forestry and Technology (Changsha, Hunan, China).

Temperature and precipitation data, which included average daily max temperature of each month (MaxT, °C), average daily min temperature of each month (MinT, °C), average daily mean temperature of each month (AveT, °C), and summed precipitation per month (Pre, mm) from 1969 to 2019 in four sample sites were obtained from Google Earth and the China Meteorological Data Sharing Service System (http://cdc.cma.gov.cn/ shishi/climate.jsp). This data was classified on a seasonal basis, into spring (Mar, Apr, May), summer (Jun, Jul, Aug), autumn (Sep, Oct, Nov), and winter (Dec, Jan, Feb) for a given year.

### Data analysis

One-way analysis of variance (ANOVA) and the Tukey-HSD test were used to analyze differences of each wood physical property among the five geographic sources, at the confidence level of 0.95. Pearson correlations were used to analyze the relationship between climatic factors and the physical properties of Chinese fir. To resolve multicollinearity in the dataset, which consisted of 19 wood property variables, the variance inflation factor (VIF) was used^62^. Stepwise linear regression was used to filter the 19 wood property variables, and find out which variables contribute the most to the wood composite index (PCA composite score value) without multicollinearity. PCA was used as a method of dimension reduction transformation. All these statistical analyses were performed in R software v3.6.2^63^.

## Supplementary information

Supplementary information.

## Data Availability

Data could be shared directly through email to corresponding author email of Prof. Dr. Xiangwen Deng (dengxw@csuft.edu.cn).
