# Supplementary material for: Variation in wood physical properties and effects of climate for different geographic sources of Chinese fir in subtropical area of China
Source: Sci Rep. 2021 Feb 25;11:4664. doi: 10.1038/s41598-021-83500-w (PMC7907201; doi:10.1038/s41598-021-83500-w)
Supplement: Supplementary file 1 — Supplementary information. [file 41598_2021_83500_MOESM1_ESM.docx]

**Variation in wood physical properties and the effects of climate for different geographic sources of Chinese fir in subtropical area of China**

**Ren You^1^ • Ninghua Zhu^2^* • Xiangwen Deng^1,3,4,^* • Jing Wang^2^ •** Fei Liu**^2^**

*^1^ Faculty of Life Science and Technology, Central South University of Forestry and Technology, Changsha 410004, Hunan Province, China*

*^2^ Faculty of forestry; Central South University of Forestry and Technology, Changsha, 410004, China*

*^3^ National Engineering Laboratory for Applied Technology of Forestry & Ecology in South China, Changsha, 410004, China*

*^4^ Huitong National Field Station for Scientific Observation and Research of Chinese Fir Plantation Ecosystem in Hunan Province, Huitong 438107, China*

******* *Correspondence: Ninghua Zhu, E-mail: [zhuninghua@yahoo.com;](mailto:zhuninghua@yahoo.com;)*

*Xiangwen Deng, E-mail: dengxw@csuft.edu.cn; Tel.: +86-731-85623458*

***Supplement***

| **Items** | **Abbreviations** | **Full Name** |
| --- | --- | --- |
| Geographic sources | FJYK-P | Normal Chinese fir of Yangkou |
|  | HNYX-T | Black-heart Chinese fir of Yongshun |
|  | HNYX-P | Normal Chinese fir of Yongshun |
|  | HNZJJ-P | Normal Chinese fir of Zhangjiajie |
|  | JXCS-R | Red-heart Chinese fir of Anfu |
| Density | WBD | wood basic density |
|  | WDD | wood all-dry density |
|  | WAD | wood air-dried density |
| Mechanical properties | MOE | modulus of elasticity |
|  | MOR | modulus of rupture |
|  | TSG | tensile strength parallel to grain |
|  | CSG | compression strength parallel to the grain |
|  | CPG.TT | compression strength perpendicular to the grain (total tensile) |
|  | CPG.TR | compression strength perpendicular to the grain (total radial) |
|  | CPG.PT | compression strength perpendicular to the grain (part tensile) |
|  | CPG.PR | compression strength perpendicular to the grain (part radial) |
| Shrinkage | TSR.RD | tangential shrinkage rate (air-dry) |
|  | RSR.RD | radial shrinkage rate (air-dry) |
|  | DDS.RD | difference dry shrinkage (air-dry) |
|  | VSR.RD | volume shrinkage rate (air-dry) |
|  | TSR.LD | tangential shrinkage rate (all-dry) |
|  | RSR.LD | radial shrinkage rate (all-dry) |
|  | DDS.LD | difference dry shrinkage (all-dry) |
|  | VSR.LD | volume shrinkage rate (all-dry) |
| Climate factors | MaxT | average daily max temperature of each month |
|  | MinT | average daily min temperature of each month |
|  | AveT | average daily mean temperature of each month |
|  | MAP | mean annual precipitation |
|  | Pre | sum precipitation of every month |
